# Quetiapine fumarate augmentation for patients with a primary anxiety disorder or a mood disorder: a pilot study

**DOI:** 10.1186/1471-244X-12-162

**Published:** 2012-09-29

**Authors:** Yi-Chih Chen, Chih-Ken Chen, Liang-Jen Wang

**Affiliations:** 1 Department of Psychiatry, Chang Gung Memorial Hospital at Keelung, Keelung, Taiwan; 2 Chang Gung University School of Medicine, Taoyuan, Taiwan

**Keywords:** *Quetiapine*, Anxiety disorders, Mood disorders with comorbid anxiety symptoms

## Abstract

**Background:**

Comorbid anxiety symptoms,in patients with a primary anxiety disorder or a mood disorder, leads to poor patient outcomes and burdens the healthcare system. This pilot study evaluated the feasibility of extended-release quetiapine fumarate (quetiapine XR) for the treatment of patients with either a primary anxiety disorder or a mood disorder with comorbid anxiety symptoms compared to a placebo, as an adjunct to antidepressant therapy.

**Methods:**

Thirty-nine patients with a diagnosis of a primary anxiety disorder or a mood disorder with comorbid anxiety symptoms were enrolled in this study. Patients with a stable dose of antidepressant therapy were randomized according to a 2:1 probability of receiving either quetiapine XR or a placebo adjunctive treatment for 8 weeks. The efficacy was assessed by the Hamilton Anxiety Rating Scale (HAM-A) and the Clinical Global Impression of severity (CGI-S) score at baseline, week 1, 4, and 8.

**Results:**

A total of 35 patients were included in this intention-to treat (ITT) population for the efficacy analysis (quetiapine XR: 22 patients; placebo: 13 patients). At week 4, statistically significant differences were observed on both the HAM-A score (p = 0.003) and the CGI-S score (p = 0.025), favouring the quetiapine XR (−13.00 ± 4.14) compared to placebo (−6.63 ± 5.42). However, no statistically significant difference was observed between the two groups with regard to changes from the baseline to week 8 on the HAM-A score (p = 0.332) or the CGI-S score (p = 0.833).

**Conclusions:**

Augmentation of antidepressant treatment with quetiapine XR did not result in clinical improvement according to the outcome measure of anxiety using the HAM-A and CGI-S scores at week 8, among the patients with either a primary anxiety disorder or a mood disorder with comorbid anxiety symptoms. However, treatment with quetiapine XR as an adjunct to antidepressant therapy appeared to provide a short-term benefit at 4 weeks. Further study is needed with a larger sample size, randomized controlled design and control of the dosage prescribed.

**Trial registration:**

Clinicaltrials.gov identifier: NCT00912535

## Background

Anxiety disorders can manifest as a primary disorder or as a comorbid symptom associated with other psychiatric disorders such as a major depressive disorder, bipolar disorder, schizophrenia, or a substance abuse disorder. Major depressive disorder (MDD) with comorbid anxiety is highly prevalent: ~85% of adults with major depression also exhibit significant anxiety symptoms [1]. Comorbid anxiety leads to more severe symptoms, decreased psychosocial functioning, a higher risk of suicide and a more chronic course, compared to a major depressive disorder alone [2,3]; in addition, it is associated with poorer and slower treatment response [1,4]. The treatment options for primary anxiety and comorbid anxiety symptoms include: selective serotonin reuptake inhibitors (SSRI), serotonin-norepinephrine reuptake inhibitors (SNRI), tricyclic antidepressants and other antidepressants. However, not all patients equally benefit from the anxiolytic effects of antidepressants.

Treatment strategies for patients with anxiety disorders and comorbid anxiety symptoms that have not responded to therapy with an antidepressant include: changing the antidepressant monotherapy, augmentation with another agent or combination therapy with another antidepressant agent [5]. Although augmentation or combination therapy is commonly used in clinical practice, evidence supporting these strategies is limited [6,7]. The benzodiazepines are frequently used to treat sleep and anxiety in addition to SSRI therapy for a major depressive disorder with comorbid anxiety; however, cognitive impairment and concerns with regard to potential abuse and/or dependency with these agents has limited their use. In clinical practice, the atypical antipsychotics are commonly used as augmentation therapy in patients presenting with a mood disorder (typically a major depressive disorder) with comorbid anxiety symptoms [8-11].

Support for investigation of atypical antipsychotics in patients presenting with a mood disorder and comorbid anxiety is based in part on preclinical studies suggesting that several atypical antipsychotics are potent 5-HT2A antagonists at low doses [12-14] and may facilitate the action of serotonin at the 5-HT1A receptor, thereby augmenting the efficacy of antidepressants [15]. In addition, certain atypical antipsychotics have other pharmacologic properties that may contribute to antidepressant effects including: α2 antagonism (risperidone), 5-HT1A agonism (aripiprazole and ziprasidone) and monoamine reuptake blockade (ziprasidone). Furthermore, there is some evidence that atypical antipsychotics are effective in the treatment of anxiety symptoms, suggesting their possible clinical utility for major depression and comorbid anxiety [16-19].

Quetiapine is known as an atypical antipsychotic with a moderate affinity for 5-HT2A serotonergic, α1-adrenergic, muscarinic and histaminergic receptors, a minor affinity for dopamine D2 and 5-HT1A receptors, and a low affinity for 5-HT2C, α2-adrenergic and D1 receptors [20]. While atypical antipsychotics have been successfully used for the treatment of mood disorders with comorbid anxiety, comprehensive data on the clinical efficacy of adjunctive antidepressant therapy with quetiapine for patients with a primary anxiety disorder or a mood disorder with comorbid anxiety has not been collected. This pilot study evaluated the feasibility of a randomized, placebo-controlled study design for testing the safety and efficacy of extended-release quetiapine fumarate (quetiapine XR) as adjunctive therapy added to antidepressants for the treatment of patients presenting with a primary anxiety disorder or a mood disorder with comorbid anxiety.

## Methods

The study was conducted using a randomized double-blind placebo-controlled design. The study was carried out at a single site and was approved by the institutional review board at the Chang Gung Memorial Hospital, Keelung, Taiwan. Written informed consent was obtained from all subjects before participation. The investigation was recorded in the clinical registry as NCT00912535.

### Patient population

For the present study, patients were randomized according to a 2:1 probability of receiving either quetiapine XR or a placebo for 8 weeks in order to minimize exposure to antidepressant monotherapy (placebo-controlled group).

For inclusion in the study the patients fulfilled all of the following criteria: (1) provision of written informed consent, (2) diagnosis of a primary anxiety disorder or a mood disorder with comorbid anxiety symptoms by the Diagnostic and Statistical Manual of Mental Disorders-Fourth Edition (DSM-IV) [21], (3) a 14-item Hamilton Anxiety Scale score (HAM-A) ≥14 [22], (4) the subject had received a single antidepressant at a therapeutic dose for at least 6 weeks, (5) male or female gender 18–65 years of age, (6) female patients of childbearing potential were using a reliable method of contraception and had a negative urine human chorionic gonadotropin (HCG) test at enrolment, (7) and were able to understand and comply with the requirements of the study and sign the informed consent.

Any of the following was regarded as a criterion for exclusion from the study: (1) pregnancy or lactation, (2) any DSM-IV Axis I disorder not defined in the inclusion criteria, (3) receiving any antipsychotic 7 days prior to entering the study, (4) patients who, in the opinion of the investigator, posed an imminent risk of suicide or a danger to self or others, (5) administration of a depot antipsychotic injection within one dosing interval (for the depot) before randomization, (6) substance dependence or substance abuse within 4 weeks prior to enrolment (except for caffeine or nicotine), as defined by DSM-IV criteria, (7) unstable or inadequately treated medical illness (e.g. congestive heart failure, angina pectoris, hypertension) as judged by the investigator, (8) and/or previous enrolment or randomization of treatment in the present study.

### Randomization

Subjects were strictly randomized sequentially. The randomization code was generated according to a randomization table, was sealed in envelopes and subsequently distributed to the study site prior to study initiation (Additional file 1).

### Blinding

Patients received one of two treatment packages according to a treatment allocated randomization number. Quetiapine XR and its matching placebo were indistinguishable in terms of appearance, smell, taste and dose regimen. Neither the investigators nor subjects knew whether the treatment package received by the patients was the study drug or placebo. AstraZeneca Ltd provided the packages.

### Study medication

Quetiapine XR was given orally at a flexible dose of 50-300 mg/day according to the judgment of the investigator, for 8 weeks, as adjunct to the antidepressant the patient was taking. The placebo was given orally, as adjunct to the antidepressant the patient was taking. Patients already receiving hypnotics or anxiolytic benzodiazepines were permitted to continue taking them at the same dose.

### Efficacy evaluations

The primary end point was the mean change from baseline to Week 8 according to the total score on the Hamilton Anxiety Scale (HAM-A) [22]. Additional efficacy evaluations included the change from baseline to all time points on the Clinical Global Impression–severity of illness (CGI-S) score [23].

### Safety and tolerability evaluations

Safety was evaluated from baseline to the end of the study by comparing clinically significant changes. The following parameters were used: (1) Abnormal Involuntary Movement Scale [24], (2) Barnes-Akathisia Rating scale [25], (3) Simpson-Angus Scale [26], (4) physical examination, (5) body weight, (6) vital signs, (7) and adverse events (AEs).

### Statistical analyses

Data analysis and summary of safety and efficacy were performed on the intention-to-treat (ITT) population; this included patients that had taken at least one dose of the study medication and had at least one evaluation for the primary efficacy endpoint, regardless of their compliance with the protocol. A last-observation-carried-forward (LOCF) analysis was applied if data were unavailable at the analysis time point. A two-sample *t*-test was used for continuous variables and the chi-square test was applied to all categorical variables. An analysis of covariance (ANCOVA) was used to test superiority of the treatment group over the placebo group for changes from baseline according to the total scores on the HAM-A and the CGI-S. All statistical tests were two-tailed, and the level of significance was set at p < 0.05.

## Results

### Disposition of patients

**Figure 1:**
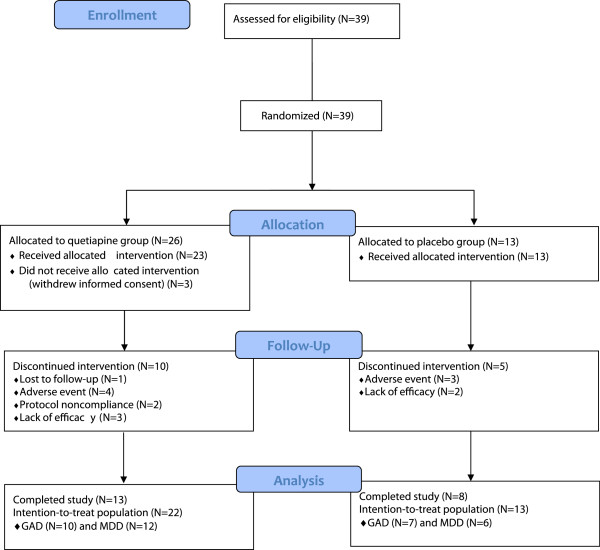
For the present study, patients were randomized with a 2:1 probability of receiving either quetiapine (N = 26), or placebo(N = 13) for 8 weeks in order to minimize exposure to a single SSRI/SNRI alone(placebo-controlled group).A total of 35 eligible patients who used the study medication and had at least one follow-up evaluation were included in the intention-to treat (ITT) population. A total of 21 patients completed the study.

The patient flow is shown in Figure 1. A total of 35 eligible patients that received study medication and had at least one follow-up evaluation were included in the intention-to-treat (ITT) population (quetiapine XR: 22 patients; placebo: 13 patients). The antidepressants that were taken by the patients included escitalopram, paroxetine, venlafaxine, duloxetine and mirtazapine. A total of 21 eligible patients (quetiapine XR: 13 patients; placebo: 8 patients) completed the study. Table 1 shows the demographic data of the ITT population. All demographic data was comparable between the quetiapine XR group and the placebo group with no statistically significant differences (p > 0.05). At baseline, the total HAM-A score, among the ITT population with LOCF, was 24.73 ± 4.45 and 27.15 ± 3.95 in quetiapine XR and placebo groups, respectively. Throughout the study, the mean percent age of study drug taken was 99.78 ± 18.74% in the quetiapine XR group, and 95.21 ± 10.61% in the placebo group. The mode and median dose of quetiapine XR was 50 mg/day, in this study.

**Table 1:** Demographic and other baseline characteristics of the ITT population

**Characteristics**	**Quetiapine XR N =** **22**	**Placebo N = 13**	***p *-value**
**Age**, years			0.057
Mean (S.D.)	42.32 (8.99)	48.57 (9.17)	
Median	40.15	47.95	
**Height**, cm			0.699
Mean (S.D.)	159.77 (5.70)	160.62 (6.91)	
Median	158.50	160.00	
**Gender**			0.832
Male	4 (18%)	2 (15%)	
Female	18 (82%)	11 (85%)	
**HAM-A**			--
Mean (S.D.)	24.73 (4.45)	27.15 (3.95)	
Median	24.00	28.00	

*ITT population* Intention-to-treat population, *HAM-A* Hamilton Anxiety Scale.

### Change from baseline to Week 8 on the total HAM-A scores

At Week 8, the mean HAM-A score was 13.15 ± 5.30 and 18.13 ± 5.69 for quetiapine XR and placebo groups, respectively; there was no statistically significant difference observed between the two groups with regard to changes from baseline to Week 8.In a subgroup analysis, there was no statistically significant difference (p = 0.175) in the HAM-A score between patients treated with quetiapine XR (N = 10) or placebo (N = 7) among the group with a primary anxiety disorder. In addition, there was no significant difference (p = 0.241)in this measure, in the patients treated with quetiapine XR (N = 12) or placebo (N = 6),among the patients with a mood disorder and comorbid anxiety symptoms. There was no significant difference in the treatment response between these two groups.

**Figure 2:**
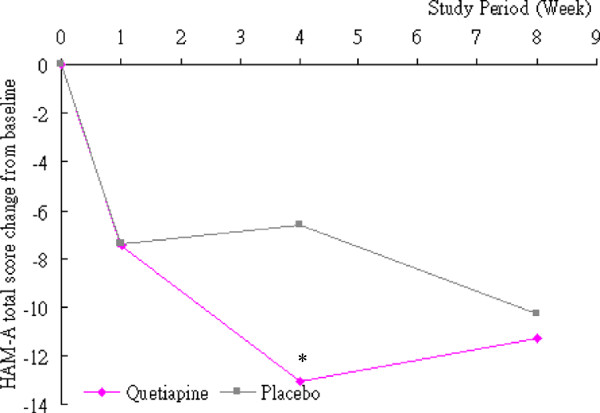
During 8 weeks of quetiapine XR augmentation to antidepressant treatment among patients with a primary anxiety disorder or a mood disorder with comorbid anxiety symptoms, a statistically significant difference was observed between the quetiapine XR group and placebo group at Week 4 compared to baseline on the total HAM-A scores (p = 0.003).However, no statistically significant difference was observed between the two groups at Week 8 compared to baseline on the total HAM-A scores.HAM-A: Hamilton Anxiety Scale.

At Week 4, however, there was an approximately two-fold change from baseline to Week 4 in the HAM-A score for patients receiving quetiapine XR (−13.00 ± 4.14) compared to those receiving placebo (−6.63 ± 5.42) [p = 0.003] (Figure 2).

### Change from baseline to Week 8 in CGI-S scores

At Week 8, the mean CGI-S score was 3.50 ± 0.86 and 3.62 ± 0.51 for the quetiapine XR and placebo groups, respectively; there was no statistically significant difference observed between the two groups with regard to changes from baseline to Week 8 on the CGI-S scores.

**Figure 3:**
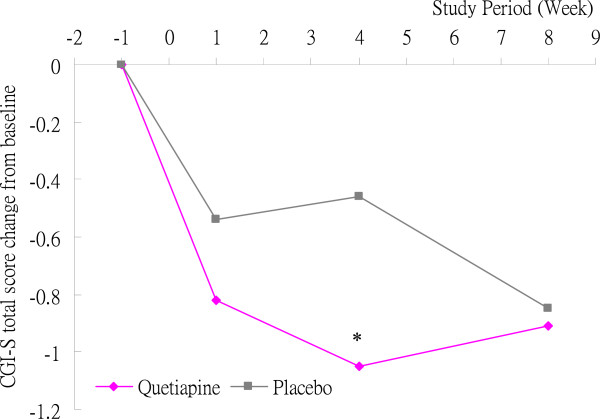
During 8 weeks of quetiapine XR augmentation to antidepressant treatment among patients with a primary anxiety disorder or a mood disorder with comorbid anxiety symptoms, a statistically significant difference was observed between the quetiapine XR group and the placebo group at Week 4 compared to baseline on the CGI-S score (p = 0.025). However, no statistically significant difference was observed between the two groupsat Week 8 compared to baseline on the CGI-S score. CGI-S: Clinical Global Impression–severity of illness.

However, at Week 4, there was up to a 2.3-fold change from baseline to Week 4 on the CGI-S score in patients receiving quetiapine XR (−1.05 ± 0.95) compared to the placebo group (−0.46 ± 0.52). A statistically significant difference was observed between the two groups in a change from baseline to Week 4in the CGI-S score (p = 0.025) (Figure 3).

### Safety and tolerability

A total of 36 patients (quetiapine XR: 23 patients; placebo: 13 patients) were included in the drug safety analysis. The rate of discontinuation due to adverse events was 17% (N = 4) in the quetiapine XR group and 23% (N = 3) in the placebo group. Throughout the treatment period, 27 patients reported 60 adverse events. There were 35 adverse events reported by 17 patients in the quetiapine XR group, and 25 adverse events reported by 10 patients in the placebo group (Table 2). Furthermore, 17 patients in the quetiapine XR group and 10 patients in the placebo group reported treatment-related adverse events. All treatment related adverse events were considered to be “Mild” or “Moderate” in severity by the investigator. None of the patients in the quetiapine XR or placebo groups experienced a serious adverse event during the treatment period. The most common adverse events reported by patients were: dry mouth, dizziness, somnolence, constipation and sedation.

**Table 2:** Summary of treatment-related events

**System organ class /Preferredterm**	**Quetiapine XR N =** **23**	**Placebo N = 13**
Subjects with at least 1 related adverse event	17 (47%)	10 (28%)
Eye disorders	0 (0%)	1 (3%)
Vision blurred	0 (0%)	1 (3%)
Gastrointestinal disorders	9 (25%)	4 (11%)
Constipation	3 (8%)	2 (6%)
Dry mouth	7 (19%)	2 (6%)
Nausea	1 (3%)	0 (0%)
Vomiting	1 (3%)	0 (0%)
Investigations	3 (8%)	4 (11%)
Blood pressure increased	0 (0%)	1 (3%)
Glycosylated haemoglobin increased	0 (0%)	1 (3%)
Weight increased	3 (8%)	3 (8%)
Metabolism and nutrition disorders	2 (6%)	2 (6%)
Increased appetite	2 (6%)	2 (6%)
Nervous system disorders	11 (31%)	5 (14%)
Dizziness	4 (11%)	1 (3%)
Headache	1 (3%)	1 (3%)
Memory impairment	0 (0%)	1 (3%)
Restless legs syndrome	0 (0%)	1 (3%)
Sedation	3 (8%)	1 (3%)
Somnolence	4 (11%)	1 (3%)
Tremor	1 (3%)	1 (3%)
Renal and urinary disorders	1 (3%)	1 (3%)
Dysuria	1 (3%)	1 (3%)
Reproductive system and breast disorders	0 (0%)	1 (3%)
Oligomenorrhoea	0 (0%)	1 (3%)
Skin and subcutaneous tissue disorders	0 (0%)	1 (3%)
Rash papular	0 (0%)	1 (3%)

At week 8, the changes in patient weight were 1.25 ± 1.87 and 2.44 ± 3.81 for the quetiapine XR and placebo groups, respectively. No statistically significant changes were observed between the two groups from baseline to week 4 and week 8. Throughout the study, one patient in the quetiapine XR group had a weight gain ≥7% at Week 8, while two patients in the placebo group had a weight gain ≥7% at Week 1, Week 4 and Week 8. The results of laboratory measurements, vital signs, physical examinations, weight change and body mass index were clinically comparable in comparisons between the two groups. Throughout the treatment period, no effect on the AIMS global severity scores was observed in the quetiapine XR or placebo groups. Moreover, there were no statistically significant differences between the two groups according to the BARS and SAS rating scales.

## Discussion

The primary outcome measures (HAM-A and CGI-S) assessed in this pilot study showed no significant differences between the quetiapine XR and placebo groups at week 8. However, treatment with quetiapine XR as an adjunct to antidepressant therapy appeared to provide a short-term benefit at 4 weeks in difficult-to-treat patients with primary or comorbid anxiety despite on-going antidepressant treatment. These results should be considered preliminary due to the limitation of a small sample size. Based on a mean change in the HAMA of 9 in the quetiapine XR arm and 4 in the placebo arm (difference = 5) and a deviation of 8 for both treatments, 35 patients in the placebo arm and 75 patients in the quetiapine XR arm would be needed to have a power of 85% at a significance level of 0.05.

A previous flexible-dose open-label pilot study, reported by Katzman et al., found that adjunctive quetiapine XR significantly reduced HAM-A scores at week 12 in patients with treatment-resistant or non-remitted GAD [27]. The results of our study showed a significant reduction in the HAM-A score at Week 8 with adjunctive quetiapine XR. However, no statistically significant difference was observed between the treatment and placebo groups. Placebo effects clearly played a significant role in the reduction of anxiety measured in this study. The failure to find a significant difference on the primary efficacy measure at week 8 could in part be explained by the small sample size as well as the heterogeneous study group. Although quetiapine monotherapy has been demonstrated to be effective, demonstrating quetiapine efficacy as adjunct therapy to antidepressant has proven difficult [28,29]. The analyses on the subsets of patients with a primary anxiety disorder and those with a mood disorder and comorbid anxiety symptoms revealed no significant difference in the treatment response between these two groups. However, these analyses lacked statistical power due to the small sample sizes after stratification.

Quetiapine XR was generally safe and well-tolerated as adjunctive treatment in patients with primary or comorbid anxiety [30,31]. In this study, EPS-related AEs were not observed with quetiapine XR during the study period. Laboratory data revealed no clinically relevant changes in glucose in the patients receiving quetiapine XR. At week 8, the changes in patient weight were 1.25 ± 1.87 and 2.44 ± 3.81 for quetiapine XR and placebo groups, respectively. However, the benign side effect profile, noted in this study, might have been due to the low dose used.

In addition to the above-mentioned limitations associated with the small sample size, other limitations of the present study include the following. It is difficult to clearly distinguish patients with a primary anxiety disorder from those with a mood disorder and comorbid anxiety symptoms. Therefore, the possibility of misclassification could not be ruled out. Regarding dosing, the dose of quetaipine XR in this study was relatively low, and the flexible dosing schedule might have confounded the results. A similar study in the future should consider a multiple arms design with different fixed doses to determine dose-dependent effects.

## Conclusion

Augmentation of antidepressant treatment with quetiapine XR did not result in clinical improvement in the outcome measures of anxiety using the HAM-A and CGI-S scores at week 8 in patients with either a primary anxiety disorder or a mood disorder with comorbid anxiety symptoms. However, treatment with quetiapine XR as an adjunct to antidepressant therapy appeared to provide a short-term benefit at 4 weeks. The results of this pilot study should be considered preliminary due to the limitations of a small sample size and the dosing strategy. A well-powered sample size and a multiple-arms design with different fixed doses to determine dose-dependent effects should be considered for future studies.

## Competing interests

In the past 3 years, YCC received lecture fees from Astra-Zeneca Ltd. CKC received research support from AstraZeneca Ltd, Lundbeck Ltd and Otsuka Taiwan Ltd. LJW has no additional disclosure to make. AstraZeneca Ltd funded this study.

## Authors' contributions

YCC and CKC contributed equally to this study and manuscript. LJW was involved in the clinical work. All authors read and approved the final manuscript.

## Pre-publication history

The pre-publication history for this paper can be accessed here:

http://www.biomedcentral.com/1471-244X/12/162/prepub

## Supplementary Material

Additional file 1 CONSORT 2010 checklist of information to include when reporting a randomised trial. Click here for file
